# 

**Published:** 1872-06

**Authors:** 


### The Fourteenth Annual Meeting

Of the Indiana State Dental Association will be held in the lecture room of the Indiana Medical College in the city of Indianapolis, commencing Tuesday, June 25th, 1872, at 2 o'clock, P. M.

The profession generally is cordially invited, and the two hundred Dentists of Indiana are especially urged to turn out in force.

SUBJECTS FOR DISCUSSION:

1st. The Essential Principles in Filling Teeth.
2d. Dr. Arthur's System of Prevention, and Treatment of Decay of the Teeth.
3d. Dietary Influences upon the Formation of the Teeth.
4th. Dental Medicines; new remedies.
5th. Dental Irregularities; best æt. for treatment.
6th. In what cases should dental pulp be destroyed.
7th. The various preparations of gold for filling.
8th. Broken points for pluggers.
9th. Base for artificial teeth, best substitute for rubber.

JOHN F. JOHNSTON, Indianapolis,
WM. L. ANDREWS, Kendallville,
E. V. BURT, Lafayette,
Executive Committee.

